# Validation of the Filipino Version of the Eustachian Tube Dysfunction Questionnaire-7 (ETDQ-7)

**DOI:** 10.7759/cureus.73363

**Published:** 2024-11-10

**Authors:** Melanie Grace Y Cruz, Kathleen T Manarang, Rubiliza D Onofre-Telan, Ma. Natividad A Almazan

**Affiliations:** 1 Otolaryngology - Head and Neck Surgery, Far Eastern University – Dr. Nicanor Reyes Medical Foundation (FEU-NRMF) Medical Center, Quezon City, PHL; 2 Otolaryngology - Head and Neck Surgery, East Avenue Medical Center, Quezon City, PHL; 3 Otolaryngology - Head and Neck Surgery, Mother Teresa of Calcutta Medical Center, San Fernando, PHL; 4 Otolaryngology - Head and Neck Surgery, Amang Rodriguez Memorial Medical Center, Marikina, PHL; 5 Otolaryngology - Head and Neck Surgery, De Los Santos Medical Center, Quezon City, PHL; 6 Otolaryngology - Head and Neck Surgery, St. Luke's Medical Center, Quezon City, PHL

**Keywords:** ent, etdq-7, eustachian tube, eustachian tube dysfunction, otology, philippines

## Abstract

Introduction

The Eustachian tube (ET) serves various functions, such as pressure regulation, mucociliary clearance, and middle ear protection. Eustachian Tube Dysfunction (ETD) pertains to a problem with the ventilatory function of the Eustachian tube and is a common condition seen in the ENT clinic. There is currently no gold standard in diagnosing ETDs, but numerous measures are being employed and studied. The Eustachian Tube Dysfunction Questionnaire 7 (ETDQ-7) is the only patient-reported outcome tool to have undergone initial validation tests. It has been translated into different languages, and studies have shown that the ETDQ-7 is a fast, reliable, and easy-to-use tool in the local setting. This tool can also be applied in the Philippine setting and may be used in conjunction with history and clinical findings to diagnose and evaluate the treatment of ETD.

Objectives

The objective of this study is to translate the Eustachian Tube Dysfunction Questionnaire-7 (ETDQ-7) into the Filipino version and validate the Filipino version of the ETDQ-7. The study also determines the prevalence of Eustachian tube dysfunction among adult Filipinos using the translated version of ETDQ-7.

Methods

A cross-sectional analytical study was conducted in the Department of Otolaryngology-Head and Neck Surgery (ORL-HNS) of a tertiary hospital. Filipinos aged 18 years and above, with and without signs and symptoms of Eustachian tube dysfunction, with intact tympanic membranes were included in the study.

Results

The Filipino version of the ETDQ-7 has good internal validity and consistency. For the test-retest reliability of the questionnaire across time, all seven questions and the total score have good reliability. It will give reliable results when repeatedly used over time.

Conclusion

The Filipino version of the ETDQ-7 is a valid and reliable tool that may serve as an adjunct in diagnosing ET dysfunction among adult Filipinos. Further studies may be needed to evaluate its use in children and patients with comorbid conditions such as ear and sinonasal pathologies.

## Introduction

The Eustachian tube (ET) is responsible for pressure regulation between the body and the atmosphere [1]. Eustachian tubes that are excessively open or patulous, baro-challenged, and those that fail to dilate properly lead to dysfunction, which may manifest in various ways. Eustachian Tube Dysfunction (ETD) pertains to a problem with the ventilatory function of the Eustachian tube and is a common condition seen in the ENT clinic [2]. A study on British adults showed the prevalence rate to be 0.9% [3]. Park et al. reported that 28.9% of patients who complained of ear fullness were diagnosed with ETDs [4]. Although its exact prevalence is unknown among Asian countries, ETDs cause a significant burden to those affected by it [1]. Because of the obscure location of the Eustachian tube, its function cannot be directly assessed. There is currently no gold standard in the diagnosis of ETDs. Still, numerous measures are being employed and studied, such as pneumatic otoscopy, tympanometry, 9-step inflation-deflation test, and video endoscopy of the nasopharyngeal end of the Eustachian tube, among others [5-8].

The Eustachian Tube Dysfunction Questionnaire 7 (ETDQ-7) is the only patient-reported outcome tool to have undergone initial validation tests [2]. It is a seven-item instrument developed and validated by McCoul et al. in 2012 [9]. The Likert-type scale questionnaire was designed to obtain a more precise disease burden and to produce formal and validated documentation of patient-reported history for the clinical record. A score of >14.5 provided 100% sensitivity and 100% specificity for subjects having ETD [9].

The ETDQ-7 was used in several studies. Van Roeyen et al. determined that the questionnaire can discriminate between normal subjects and those with baro-challenged ETDs [10]. Likewise, it was used in Han's study, which demonstrated that Dynamic Slow Motion Video Endoscopy (DSVE) combined with the ETDQ-7 correlates well with a Eustachian tube obstruction [2]. The questionnaire was translated into Korean [2], Turkish [11], and German [12]. In these studies, patients with obstructed Eustachian tubes were found to have mean scores of 18.58 to 24.7 and 8.67 to 12.7 among healthy subjects [2,11,12].

This study aimed to translate the Eustachian Tube Dysfunction Questionnaire-7 (ETDQ-7) to Filipino and validate the questionnaire as an assessment tool for Eustachian tube function among adult Filipinos. Using the translated questionnaire, the study also aimed to determine the prevalence of Eustachian tube dysfunction among adult Filipinos.

## Materials and methods

Methods

Study Design and Setting

The cross-sectional analytical study was conducted from August 2017 to October 2019 at the Department of Otolaryngology-Head and Neck Surgery (ORL-HNS) of East Avenue Medical Center, a tertiary referral center in the Philippines.

The primary investigator sought permission from Dr Vijay Anand, one of the inventors and the correspondent author for the ETDQ7 tool, to translate it into the Filipino language and perform validation.

Before the commencement of the study, approval from the Institutional Ethics Review Board of East Avenue Medical Center was obtained.

Subjects

Inclusion and Exclusion Criteria

All adult Filipinos aged 18 and above, who consulted in the ORL-HNS outpatient clinic during the study period, and who could give verbal and written consent were recruited regardless of their Eustachian tube status.

Patients who have perforated tympanic membranes, with or without discharge, recent upper respiratory infection, nasal polyposis, presence of cleft lip and/or palate; those who have mental illnesses; and those who have undergone surgical and/or interventional treatment for Eustachian tube dysfunction were excluded.

Materials and methods

Phase I - Translation

Twenty adults from the same pool as the target population were tasked to answer and review the translated questionnaire's consensus version to determine which items could not be fully understood by the potential respondents. They were allowed to suggest replacement or substitute terminologies that will be considered in developing the pre-final draft of the translated tool. The same respondents performed a second review of the pre-final draft that has undergone the back translation and expert panel review for final evaluation of the understandability of the translated instrument.

Phase II - Validation

The participants were asked to fill out a general information sheet of demographic data and complete the translated Eustachian Tube Dysfunction Questionnaire 7 (ETDQ-7). The primary investigators assisted in the administration of the questionnaires.

The Instrument

The Eustachian Tube Dysfunction Questionnaire 7 (ETDQ-7) is a seven-item instrument developed and validated by McCoul et al. in 2012 [9]. This Likert scale questionnaire was designed to obtain a more precise estimate of disease burden and create a structured symptom documentation (Table 1).

**Table 1:** The Seven-Item Eustachian Tube Dysfunction Questionnaire (ETDQ-7), Original English Version

Over the past 1 month, how much has each of the following been a problem for you?	No Problem		Moderate Problem			Severe Problem	
1. Pressure in the ears?	1	2	3	4	5	6	7
2. Pain in the ears?	1	2	3	4	5	6	7
3. A feeling that your ears are clogged or “under water”?	1	2	3	4	5	6	7
4. Ear symptoms when you have a cold or sinusitis?	1	2	3	4	5	6	7
5. Crackling or popping sounds in the ears?	1	2	3	4	5	6	7
6. Ringing in the ears?	1	2	3	4	5	6	7
7. A feeling that your hearing is muffled?	1	2	3	4	5	6	7

Data collection

Phase I - Translation

1. Forward translation: Two bilingual Filipinos translated the questionnaire from English to Filipino. One of the translators was an otorhinolaryngologist who was familiar with the area of research, and the other was a Filipino professor who was unfamiliar with the subject matter.

2. Synthesis of the translation: The differences between the two translated versions were reconciled to create the consensus version. This task was assigned to a group of translators, otorhinolaryngologists, and otologists who agreed on the terminologies that should be used in the questionnaire. A team comprising five patients was asked to review the consensus version. They were also given feedback forms.

3. Back translation: The Filipino consensus version was revised according to the feedback given by the five patients. Back translation to the original language by another native translator was performed. The primary investigators reviewed this version.

4. Review: The ETDQ-7 pre-final version was developed for preliminary studies by an expert panel comprising otolaryngologists and otologists. This was sent to the original author.

Phase II - Validation and Retest Reliability

All patients underwent a thorough medical history and an otorhinolaryngological examination, including a tympanogram and pneumatic otoscopy. Following that, the patients were divided into two groups:

1. Control group: Patients who fit the inclusion and exclusion criteria without ETD complaints, and normal pneumatic otoscopy and tympanometry.

2. ETD group: Patients who fit the inclusion and exclusion criteria with clinical symptoms of Eustachian tube dysfunction (having at least two of the following in at least one ear in the last month: ear fullness or ear pain, hearing loss or muffling, middle ear effusion, presence of tinnitus).

One hundred eighty-two participants who met the inclusion criteria were recruited consecutively. They were given the final version of the Filipino ETDQ-7 (Table 2). The first 35 respondents who were given the questionnaire also underwent re-evaluation of their symptoms. Responses were encoded and statistically analyzed for validity and test-retest reliability.

**Table 2:** The Seven-Item Eustachian Tube Dysfunction Questionnaire (ETDQ-7), Translated Filipino Version

INSTRUKSIYON: Bilugan ang bilang na naaayon sa iyong nararamdaman. Kung walang nararamdaman sa mga nasabing simtomas, bilugan ang 1. Sagutan lahat ng katanungan. Huwag mag-iwan ng blanko.							
Sa nakalipas na isang buwan, gaano ka naapektuhan ng mga sumusunod:	Walang Problema		Kaunting Problema			Matinding Problema	
1. Pakiramdam na parang may nakadiin o puno ang tenga?	1	2	3	4	5	6	7
2. Masakit ang loob ng tenga?	1	2	3	4	5	6	7
3. Pakiramdam ay barado ang tenga o parang may tubig?	1	2	3	4	5	6	7
4. May problema sa tenga kung may sipon o “sinusitis”	1	2	3	4	5	6	7
5. May naririnig na parang pumuputok o “popping sound” sa loob ng tenga?	1	2	3	4	5	6	7
6. May ugong na naririnig?	1	2	3	4	5	6	7
7. Malabo o hindi maganda ang kaledad ng tunog na naririnig?	1	2	3	4	5	6	7

Phase III - Determining the Prevalence of ETD

In addition to the ETDQ-7 responses, the demographic and clinical information of the 182 participants were gathered during the initial screening. Data obtained included age, gender, comorbidities, associated symptoms during the consultation, and other findings during otoscopy and pneumatic otoscopy.

Data and statistical analysis

Descriptive statistics included mean, standard deviation, frequency, and percentages. Cronbach's alpha analysis was used to determine the internal consistency of the respondents' responses on the ETDQ-7 during the initial test and retest. The Wilcoxon signed-rank test was used to assess the consistency of the respondents' responses in ETDQ-7 items from the initial test to the retest. The Pearson r coefficient was used to determine the translated tool's test-retest reliability over time.

The independent T-test was used to determine differences in age and ETDQ-7 scores between groups (male vs female). The Fischer exact test was used to determine the relationships between otoscopy findings and each question's score. No missing values were replaced or estimated. At the 0.05 level of significance, the null hypothesis was rejected. STATA 13.1 (StataCorp LLC, College Station, TX, USA) was used for data analysis, and MS Excel (Microsoft® Corp., Redmond, WA, USA) was used for data encoding.

## Results

Part 1. Validation of the translated Filipino version of ETDQ-7

Analysis of the first 35 subjects during their initial test showed that questions 1, 3, 4, 5, and 6 have good consistency, while questions 2 and 7 have excellent consistency (Table 3). For the retest, all seven questions have a good consistency (Table 3). These findings demonstrate that the translated questionnaire has a high internal consistency. However, when comparing the consistency between initial and retest responses, only the total score had significant consistency (Table 4). For the test-retest reliability of the questionnaire across time, all seven questions and the total score have good reliability. It will give reliable results when repeatedly used over time (Table 5).

**Table 3:** Internal consistency of the responses during the initial test and retest of the respondents on Filipino translated version of ETDQ-7 (n=35)

Item	Valid observation	Cronbach's alpha	Verbal interpretation
Initial test			
Question 1	35	0.8884	Good consistency
Question 2	35	0.9020	Excellent consistency
Question 3	35	0.8894	Good consistency
Question 4	35	0.8998	Good consistency
Question 5	35	0.8869	Good consistency
Question 6	35	0.8972	Good consistency
Question 7	35	0.9068	Excellent consistency
Retest			
Question 1	35	0.8918	Good consistency
Question 2	35	0.8803	Good consistency
Question 3	35	0.8766	Good consistency
Question 4	35	0.8902	Good consistency
Question 5	35	0.8760	Good consistency
Question 6	35	0.8780	Good consistency
Question 7	35	0.8792	Good consistency

**Table 4:** Consistency of the responses from initial test to retest (n=35)

ETDQ-7 items	Initial test	Retest	P-value
	Median (IQR)		
Question 1	1 (1 to 3)	1 (1 to 2)	0.077
Question 2	1 (1 to 2)	1 (1 to 1)	0.176
Question 3	1 (1 to 3)	1 (1 to 2)	0.426
Question 4	1 (1 to 3)	1 (1 to 3)	0.401
Question 5	1 (1 to 3)	1 (1 to 3)	0.967
Question 6	2 (1 to 4)	2 (1 to 4)	0.063
Question 7	2 (1 to 4)	1 (1 to 4)	0.090
Total score	13 (9 to 19)	12 (9 to 15)	0.048

**Table 5:** Test-retest reliability from initial test to retest (n=35)

ETDQ-7 items	Pearson r coefficient	interpretation	P-value
Question 1	.814	Good reliability	< .001>
Question 2	.877	Good reliability	< .001>
Question 3	.840	Good reliability	< .001>
Question 4	.788	Good reliability	< .001>
Question 5	.857	Good reliability	< .001>
Question 6	.840	Good reliability	< .001>
Question 7	.879	Good reliability	< .001>
Total score	.930	Excellent reliability	< .001>

Of the 182 subjects, 37.91% were male and 62.09% were female, with a mean age of 39.12+16.26 years. The age difference between males and females at 40.06+15.92 and 38.55+16.43, respectively was insignificant (p>0.05) (Table 6).

**Table 6:** Demographic and clinical characteristics of the study population *P-values calculated using an independent T-test for data on the age of male and female subjects.

Characteristic	Total	Male	Female	p-value
N	182	69 (37.91%)	113 (62.09%)	
Age (in years)	39.12+16.26	40.06+15.92	38.55+16.43	0.3738*
Comorbidities/signs and symptoms:				
Allergic Rhinosinusitis	9	4	5	
Laryngopharyngeal reflux (LPR)	4	3	1	
Dizziness	3	2	1	
Dizziness with LPR	1	0	1	
Ear Findings:				
Normal otoscopy findings	134	44 (32.83)	90 (67.16)	
Abnormal otoscopy findings (at least one ear)	23	17 (73.91)	6 (26.09)	
Normal pneumatic otoscopy findings	152	59 (38.82)	93 (61.18)	
Abnormal pneumatic otoscopy findings (at least one ear)	4	1 (25)	3 (75)	

Only a few patients who came in for consultation complained of accompanying signs and symptoms. Of the 157 ears examined via otoscopy, 14.64% had abnormal ear findings in at least one ear; out of the 156 ears tested via pneumatic otoscopy, 2.56% had abnormal tympanic membrane movement in at least one ear (Table 6).

Based on the validated questionnaire, out of the 182 subjects, 63 (34.62%) had scores higher than 14 (out of 49), indicating the presence of Eustachian tube dysfunction.

Between genders, males scored questions 1, 2, and 7 significantly higher than females (P<0.05). The male respondents found pressure in the ear (Q1), pain in the ears (Q2), and feeling of muffled hearing (Q7) to be problematic for them (Table 7).

**Table 7:** Comparison of scores in the Filipino translated ETDQ-7 between male and female subjects (n=185) P-value computed using independent t-test; significance P <0.05

ETDQ-7 items	Male (n=69)	Female (n=113)	P-value*
	Mean + SD		
Question 1	2.35+1.77	1.89+1.42	0.0065
Question 2	2.03+1.70	1.64+1.39	0.0171
Question 3	2.33+1.77	1.99+1.63	0.0574
Question 4	1.99+1.56	2.00+1.57	0.9514
Question 5	1.93+1.66	1.81+1.46	0.4645
Question 6	2.28+1.84	2.23+1.78	0.7923
Question 7	2.43+1.85	2.07+1.62	0.049
Total score	15.33+9.38	13.65+8.41	0.0728

There is a significant relationship between normal otoscopic findings and the scales for "no problem" (1-2) and "moderate problem" (3-6) in questions 1 (pressure in the ears), 2 (pain in the ears), 3 (feeling of clogged ears or underwater), and 7 (feeling of hearing muffled sounds). This means that individuals may feel symptoms of mild to moderate Eustachian tube dysfunction despite having normal otoscopic findings (Table 8).

**Table 8:** Association between responses on each question and otoscopic findings (n=157) *Fischer exact test was utilized, with significance at P<0.05.

Question	Scale	Normal otoscopy (n=134)	Abnormal otoscopy (at least one ear) (n=23)	P-value*
1	No problem	108	8	<0.0001
	Moderate to severe problem	26	15	
2	No problem	120	15	<0.005
	Moderate to severe problem	14	8	
3	No problem	108	8	<0.0001
	Moderate to severe problem	26	15	
4	No problem	99	14	0.21485
	Moderate to severe problem	35	9	
5	No problem	109	14	0.051
	Moderate to severe problem	25	9	
6	No problem	98	13	0.5606
	Moderate to severe problem	36	10	
7	No problem	103	9	0.0006
	Moderate to severe problem	31	14	

## Discussion

Patient-reported outcome measures (PROMs) are questionnaire-based tools that measure patient-centered outcomes such as symptoms and quality of life. If well-designed, they are repeatable and quantitative, providing advantages over clinical history-taking. Disease-specific PROMs are particularly useful when a gold standard objective test does not exist. The 7-Item Eustachian Tube Dysfunction Questionnaire (ETDQ-7) has undergone preliminary validation trials and has been translated into several languages. It is now used as an outcome measure in clinical and research settings [13].

Between genders, males scored questions 1, 2, and 7 significantly higher than females (P<0.05). The male respondents found pressure in the ear (Q1), pain in the ears (Q2), and feeling of muffled hearing (Q7) to be problematic for them.

There is a significant relationship between normal otoscopic findings and the scales for "no problem" (1-2) and "moderate problem" (3-6) in questions 1 (pressure in the ears), 2 (pain in the ears), 3 (feeling of clogged ears or underwater), and 7 (feeling of hearing muffled sounds). This means individuals may feel symptoms of mild to moderate Eustachian tube dysfunction despite having normal otoscopic findings.

According to Teixeira et al., symptoms such as ear pain, tinnitus, and muffled hearing can also be present in various trigeminal nerve and inner ear pathologies, so these questions do not help distinguish cases that are not related to ETD. Muffled hearing and clogged ears are symptoms that are very similar, introducing redundancy into the questionnaire and scoring system. They concluded that while the ETDQ-7 had a high correlation with symptoms, it was only moderately associated with an objective measure of ET function [14]. The questionnaire was also unable to distinguish between patients suffering from obstructive and patulous ETD. Earlier assessments of the ETDQ-7 appear to have significantly overestimated its accuracy as a diagnostic tool due to the use of healthy volunteer controls and those with unrelated pathology. Although ETDQ-7 is not critical in diagnosing ETD, it appears responsive to changes in ET symptoms. It then retains a vital role in quantifying the presence and severity of ETD-related symptoms, making them helpful in assessing change in ETD symptoms after an intervention [13].

Only a few participants in this study reported accompanying signs and symptoms during their consultations. The most common comorbidity among test subjects was allergic rhinitis. According to Spector's research, atopic individuals have a higher incidence of ET dysfunction after nasal allergen challenge than normal subjects. This association has also been observed. Children with allergies have more ET dysfunction during peak ragweed and mold season than at other times. In adults with allergic rhinitis, pretreatment with an antihistamine and/or decongestant protects against ET obstruction caused by an allergen challenge [15].

Many translated and validated versions of the ETDQ-7 include Brazilian-Portuguese, German, Mandarin, and Turkish. A locally translated version is beneficial to patients and doctors. Patients are evaluated better because of the more accurate representation of their subjective complaints. Clinicians can also assess symptom severity more accurately, allowing for more effective treatment plans. Lastly, the ETDQ-7 takes a few minutes to complete and may be conveniently administered in the clinical setting [16].

To our knowledge, this is the first Filipino version of the ETDQ-7. The authors of this study suggest that the translated Filipino version of the ETDQ-7 can be used in conjunction with clinical and physical findings to aid in the diagnosis of ET dysfunction. It is a quick, dependable, and simple-to-use tool that could be valuable in the clinical setting.

## Conclusions

There is currently no gold standard for diagnosing Eustachian tube dysfunction. A correlation of clinical symptoms, physical exam findings, and various diagnostic tests may be used to evaluate ETD. The Filipino version of the ETDQ-7 is a valid and reliable tool that can aid in diagnosing ET dysfunction in adult Filipinos. Further research may be needed to evaluate its use in children and patients with comorbid conditions such as ear and sinonasal pathologies.
